# *Blautia*-PTGS1 co-occurrence in prolactinomas: potential implications for tumor microenvironment and invasiveness

**DOI:** 10.1210/jendso/bvag085

**Published:** 2026-04-07

**Authors:** Zhen Ye, Jiayu Yang, Lijin Ji, Rui Han, Yifei Yu, Zhao Ye, Xufang Gu, Boyu Cai, Chenxing Ji, Nidan Qiao, Zhengyuan Chen, Zengyi Ma, Long Chen, Yankai Liao, Chengzhang Shi, Wenqiang He, Xiaoyun Cao, Xiang Zhou, Xuefei Shou, Yongfei Wang, Zhaoyun Zhang, Hongying Ye, Yichao Zhang, Qilin Zhang

**Affiliations:** Department of Neurosurgery, Huashan Hospital, Fudan University, Shanghai 200000, China; National Center for Neurological Disorders, Shanghai 200000, China; Department of Radiology, Huashan Hospital, Fudan University, Shanghai 200000, China; Department of Endocrinology and Metabolism, Huashan Hospital, Fudan University, Shanghai 200000, China; Department of Neurosurgery, Huashan Hospital, Fudan University, Shanghai 200000, China; National Center for Neurological Disorders, Shanghai 200000, China; Department of Endocrinology and Metabolism, Huashan Hospital, Fudan University, Shanghai 200000, China; Department of Neurosurgery, Huashan Hospital, Fudan University, Shanghai 200000, China; National Center for Neurological Disorders, Shanghai 200000, China; Department of Neurosurgery, Huashan Hospital, Fudan University, Shanghai 200000, China; National Center for Neurological Disorders, Shanghai 200000, China; Department of Neurosurgery, Huashan Hospital, Fudan University, Shanghai 200000, China; National Center for Neurological Disorders, Shanghai 200000, China; Department of Neurosurgery, Huashan Hospital, Fudan University, Shanghai 200000, China; National Center for Neurological Disorders, Shanghai 200000, China; Department of Neurosurgery, Huashan Hospital, Fudan University, Shanghai 200000, China; National Center for Neurological Disorders, Shanghai 200000, China; Department of Neurosurgery, Huashan Hospital, Fudan University, Shanghai 200000, China; National Center for Neurological Disorders, Shanghai 200000, China; Department of Neurosurgery, Huashan Hospital, Fudan University, Shanghai 200000, China; National Center for Neurological Disorders, Shanghai 200000, China; Department of Neurosurgery, Huashan Hospital, Fudan University, Shanghai 200000, China; National Center for Neurological Disorders, Shanghai 200000, China; Department of Neurosurgery, Huashan Hospital, Fudan University, Shanghai 200000, China; National Center for Neurological Disorders, Shanghai 200000, China; Department of Neurosurgery, Huashan Hospital, Fudan University, Shanghai 200000, China; National Center for Neurological Disorders, Shanghai 200000, China; Department of Neurosurgery, Huashan Hospital, Fudan University, Shanghai 200000, China; National Center for Neurological Disorders, Shanghai 200000, China; Department of Neurosurgery, Huashan Hospital, Fudan University, Shanghai 200000, China; National Center for Neurological Disorders, Shanghai 200000, China; Department of Neurosurgery, Huashan Hospital, Fudan University, Shanghai 200000, China; National Center for Neurological Disorders, Shanghai 200000, China; Department of Neurosurgery, Huashan Hospital, Fudan University, Shanghai 200000, China; National Center for Neurological Disorders, Shanghai 200000, China; Department of Neurosurgery, Huashan Hospital, Fudan University, Shanghai 200000, China; National Center for Neurological Disorders, Shanghai 200000, China; Department of Endocrinology and Metabolism, Huashan Hospital, Fudan University, Shanghai 200000, China; Department of Endocrinology and Metabolism, Huashan Hospital, Fudan University, Shanghai 200000, China; Department of Neurosurgery, Huashan Hospital, Fudan University, Shanghai 200000, China; National Center for Neurological Disorders, Shanghai 200000, China; Department of Neurosurgery, Huashan Hospital, Fudan University, Shanghai 200000, China; National Center for Neurological Disorders, Shanghai 200000, China; Neuroendocrine Unit, Massachusetts General Hospital and Division of Endocrinology, Diabetes and Hypertension, Brigham and Women's Hospital, Harvard Medical School, Boston, MA 02114-2696, USA

## Abstract

**Context:**

Prolactinomas are the most common functional pituitary adenomas. While altered gut microbiota has been observed in prolactinomas, whether these changes influence tumor behavior remains unclear. Our study integrated multi-omics data to investigate potential associations between gut microbiota composition and tumor characteristics in prolactinomas, as well as to explore potential molecular mechanisms and therapeutic pathway targets.

**Methods:**

Through 16S rRNA and mRNA sequencing of 10 paired patients, we identified *Blautia*-invasiveness associations and PTGS1 involvement in invasive heterogeneity of prolactinomas. We validated findings using large-cohort multi-omics datasets and evaluated PTGS1 as a therapeutic target in patient-derived organoids.

**Results:**

*Blautia* abundance in the gut microbiota of patients with prolactinomas correlated with tumor invasiveness. Integrating tumor transcriptomic data, we observed a positive correlation between *Blautia* abundance and PTGS1 expression. We leveraged previously published large-cohort multi-omics datasets and confirmed that PTGS1 is markedly upregulated in prolactinomas, with higher expression in highly invasive tumors. Differential expression analysis of tumor mRNA-seq revealed that PTGS1-high tumors showed enrichment of extracellular matrix remodeling and immune-related pathways associated with tumor invasiveness. Finally, targeting PTGS1 with the selective inhibitor Tenidap in patient-derived prolactinoma organoids suppressed organoid proliferation and prolactin secretion.

**Conclusion:**

We identified a correlation between *Blautia* abundance and PTGS1 expression in prolactinomas, with PTGS1-high tumors showing enrichment of tumor microenvironment remodeling and immune-related pathways. These findings suggest a potential *Blautia*-PTGS1-tumor microenvironment association in prolactinomas that requires further mechanistic investigation to establish causality.

**Keywords** Prolactinoma, Multi-omics, Blautia, Prostaglandin endoperoxide synthase 1, Tumor environment

Pituitary neuroendocrine tumors (PitNETs) are among the most common types of intracranial neoplasms, accounting for 10-20% of all cases. Approximately two-thirds of PitNETs are functional, secreting hormones such as prolactin (PRL), growth hormone (GH), and adrenocorticotropic hormone [1]. Prolactinomas, the most prevalent type of functional PitNETs, originate from lactotroph cells in the anterior pituitary gland and account for approximately 50% of all PitNET cases [2, 3]. First-line medical intervention with dopamine agonists (DAs), such as bromocriptine and cabergoline, can alleviate symptoms, normalize hyperprolactinemia, and reduce tumor size in the majority of patients [4]. Despite favorable response rates to DAs in most micro-prolactinomas, the emergence of DAs unresponsiveness or intolerance necessitates surgical intervention for invasive prolactinomas. There is a critical need to elucidate the molecular drivers of invasive prolactinomas which are characterized by larger tumor volume, higher Ki-67 index, and cavernous sinus invasion [5, 6].

The tumor microenvironment (TME) is a complex milieu in which interactions among cellular and noncellular components regulate tumor behavior and contribute to increased tumor invasiveness [7]. In prolactinomas, extensive infiltration of tumor-associated macrophages (TAMs) alters tumor biology by suppressing immune responses, promoting vascularization, and remodeling the extracellular matrix (ECM) [8-11]. The secretome of tumor-associated fibroblasts (TAFs) and the immune signatures of lymphocytes also play pivotal roles in determining the invasiveness of PitNETs [12-14]. Extracellular matrix can also contribute to the invasiveness and treatment resistance of PitNETs through the interplay between matrix metalloproteinases and tissue inhibitors of metalloproteinases [15]. Transcriptomic heterogeneity among PitNETs subtypes shapes the TME architecture, orchestrating key pathological processes such as recruitment of TAMs and TAFs and the remodeling of ECM components [9].

Prolactin has been shown to play a significant role in inflammatory processes by stimulating cytokine secretion from macrophages and interferon production from lymphocytes [16-20]. Therefore, the oversecretion of prolactin in prolactinoma patients may contribute to alterations in inflammatory factors and the tumor immune microenvironment. Prostaglandin endoperoxide synthase 1 (PTGS1), also known as cyclooxygenase-1 (COX-1), is a critical enzyme in prostaglandin biosynthesis that catalyzes the synthesis of prostaglandin H2, a precursor for pro-inflammatory and pro-angiogenic mediators such as thromboxane A2 (TXA2) and prostaglandin E2 (PGE2) [21]. Unlike PTGS2, PTGS1 is regarded as an inherent housekeeping enzyme in various organs and tissues. PTGS1 activation is implicated in macrophage and monocyte differentiation, inflammatory responses, cell proliferation, and fatty acid metabolism during tumor progression [22, 23]. The expression of PTGS1 can be modulated by selective inhibitors or probiotics [24]. Researches on probiotics and gut microbiota have demonstrated indicated that microbiota–host interactions can modulate systemic inflammation [25]. Pituitary neuroendocrine tumors frequently disrupt hypothalamic-pituitary homeostasis through mass effects, leading to dysregulation of pituitary hormone secretion-particularly in functional subtypes, where hormone hypersecretion predominates. This endocrine disturbance is associated with significant alterations in gut microbiota, as demonstrated by multi-cohort studies reporting distinct microbial signatures in PitNET patients compared to healthy controls [26, 27]. Current studies investigating the interactions PitNETs and gut microbiota interactions remain largely descriptive, focusing primarily on cataloging compositional shifts and associated metabolic and systemic dysfunctions. A fundamental knowledge gap remains regarding whether, and by what mechanisms, distantly located gut microbes actively regulate tumor transcriptional changes and biological behavior.

Here, we integrated multi-omics data from prolactinoma patient cohorts to systematically investigate associations between gut microbiota composition and tumor transcriptional profiles. We identified a positive correlation between *Blautia* enrichment in the gut microbiota and elevated tumor PTGS1 expression. PTGS1 was specifically upregulated in prolactinomas relative to other pituitary neuroendocrine tumors and demonstrated significant association with tumor microenvironment-related pathways implicated in prolactinoma invasiveness.

## Materials and methods

### Cohorts

Two clinical cohorts of prolactinoma patients were included in our study: Firstly, a paired cohort for analysis of fecal microbiota and tumor mRNA sequencing were recruited. Inclusion required a preoperative diagnosis of prolactinoma at Huashan Hospital, Department of Neurosurgery, based on standard criteria: (1) serum prolactin >5× the upper limit of normal (ULN, >200 ng/mL); (2) a sellar mass on MRI; and (3) compatible symptoms (eg, amenorrhea, galactorrhea, headache, infertility, mass effect on adjacent neurovascular structures, premature ejaculation, erectile dysfunction, and/or hypogonadism). Postoperative pathology confirmed the diagnosis according to the 2022 WHO Classification of Neuroendocrine Neoplasms [28]. Additional exclusion criteria for fecal sampling were: (1) antibiotics and/or probiotics within 6 months; (2) history of gastrointestinal tumors and/or inflammatory diseases; (3) immunosuppressive drugs within 6 months.

Cavernous sinus invasion was evaluated using the Knosp classification based on coronal T1-weighted contrasted imaging [29]: Knosp 0 when prolactinoma is medial to medial tangent; Knosp 1 if prolactinomas extend to the space between the medial tangent and the intercarotid line; Knosp 2 when prolactinomas extends to the space between the intercarotid line and the lateral tangent; Knosp 3 if prolactinomas extends lateral to the lateral tangent; and Knosp 4 with a complete encasement of intracavernous ICA. In this study, Knosp 0-2 prolactinomas were considered as low-invasiveness (LI) and Knosp 3-4 prolactinomas were considered as high-invasiveness (HI).

Healthy controls for Cohort 1 underwent 16S rRNA sequencing and were frequency-matched on general demographics from a large cohort maintained by the Department of General Surgery, Shanghai Tenth People's Hospital, School of Medicine, Tongji University [30]. Another cohort to validate the mechanism for PTGS1 in regulating the invasiveness in prolactinoma included: newly diagnosed prolactinoma patients with tumor samples collected for mRNA sequencing, enrolled preoperatively at the same center using the same diagnostic criteria, with postoperative pathological confirmation per the 2022 WHO classification.

### Postoperative remission criteria and DAs response and resistance criteria

2023 Pituitary Society Consensus definitions regarding postoperative remission and DAs response and resistance assessment was utilized in this study [3]. Specifically, preoperative DAs response was defined as prolactin normalization (<1.0×ULN) or ≥30% tumor shrinkage after standard-dose DAs (bromocriptine: 7.5-10 mg/day; cabergoline: 2.0 mg/week) for over 6 months; DAs resistance was defined as failure to achieve either criterion. Early assessment at 3 months (prolactin normalization and/or >25% tumor reduction) predicted long-term response. Postoperative remission was defined as biochemical remission (serum prolactin <1.0×ULN measured after 6 weeks post-surgery) and/or complete clinical symptom resolution (galactorrhea cessation, sustained gonadal recovery ≥6 months). Postoperative DAs response was assessed at 3-6 months as tumor stabilization and prolactin normalization.

### Fecal sample collection and DNA extraction

After admission to the ward, prolactinoma patients were required to fast overnight, and paired fecal and blood specimens were obtained the following morning before any medical intervention. Community-based healthy controls were sampled using the same procedures. Stool was collected in sterile containers (SARSTEDT, Germany; cat. 80.734.311), transported on ice at 4 °C to the laboratory within 2 hours, and promptly stored at −80 °C until sample processing. All fecal collections were performed by a single trained operator. Microbial DNA was isolated using a modified workflow derived from the QIAamp Fast DNA Stool Mini Kit (Qiagen, Germany). In brief, ∼200 mg of stool was combined with 1 mL InhibitEX Buffer and an appropriate quantity of 0.5-mm glass beads (Qiagen), then vigorously agitated on a FASTPREP-24 homogenizer (Aosheng Biotech, China) until fully lysed. Subsequent purification followed the manufacturer's protocol. DNA yield and quality were assessed with a NanoDrop 2000 spectrophotometer (Thermo Scientific, USA) and a Qubit 2.0 fluorometer (Invitrogen, USA).

### 16S rRNA sequencing data processing

After demultiplexing, reads with barcodes and primers removed were evaluated for length and base quality. 16S amplicons were retained if they were 250-500 bp, had a mean Phred score of at least 30 (Q30), and contained no more than 1 ambiguous nucleotide. Tag counts were tallied, duplicates were collapsed, and only tags occurring more than once were carried forward for Operational Taxonomic Units (OTU) construction, with 1 representative sequence per OTU. OTUs were generated at 97% similarity using UPARSE, and chimeras were detected and filtered with USEARCH (v7.0.1090). Taxonomic annotation of representative sequences was performed with the RDP Classifier (https://zenodo.org/records/10367203) against the RDP database at a confidence cutoff of 0.8 OTU tables and downstream alpha- and beta-diversity metrics were produced with QIIME (v1.9.1) Python workflows. Genus-level differential abundance was assessed using the Wilcoxon rank-sum test.

### Tumor sample collection and DNA extraction

Fresh tumor fragments were immediately frozen in liquid nitrogen under RNase-free conditions and stored at −80 °C until RNA/protein extraction. Total RNA was isolated from ∼5 mg of cryo-pulverized PitNET tissue or APG using TRIzol (Invitrogen) followed by cleanup on RNeasy MinElute columns (Qiagen). RNA quantity and integrity were assessed with an Agilent 2100 Bioanalyzer and a Thermo Fisher NanoDrop. Approximately 500 ng of high-quality RNA per sample was used for library construction.

RNA-seq libraries were prepared with Ribo-off® rRNA Depletion Kit (H/M/R) (Vazyme #N406) and VAHTS® Universal V6 RNA-seq Library Prep Kit for Illumina (#N401-NR604), with unique index barcodes assigned to each sample. Sequencing was performed on an Illumina platform to generate 150-bp paired-end reads.

### mRNA-seq data analysis

Read quality for all PitNET libraries was assessed with FastQC v0.11.9. Adapters and low-quality sequence of raw data (Fastq) were trimmed using fastp v0.22.0, and reads ≥75 bp were retained as clean reads for analysis. Clean reads were aligned to the human reference genome (hg19) with STAR v2.4.2a using default settings, and gene models were annotated with GENCODE v19. Gene and transcript abundances were quantified with RSEM v1.2.29 (estimate-rspd enabled; other parameters default). Expression levels were reported as FPKM, and transcripts with FPKM > 1 were kept for downstream analyses.

### GSEA analysis

Gene set enrichment analysis (GSEA) was conducted using the GSEA software (https://www.gsea-msigdb.org/gsea/index.jsp). Background gene sets were obtained from MSigDB v7.4 (https://data.broadinstitute.org/ gsea-msigdb/msigdb/release/7.4/), including KEGG, Reactome, Gene Ontology (GO), and HALLMARK collections.

### Organoid culture in Matrigel

Fresh tumor tissues were processed within 2 hours of collection. Samples were washed with cold phosphate-buffered saline to remove blood and debris, then mechanically dissociated into small fragments using sterile scissors. Where indicated, enzymatic digestion was performed using type IV collagenase or a combination of collagenase and DNase I to facilitate dissociation into single cells or small aggregates. The digested suspension was filtered through a 100 μm cell strainer to remove undigested material, and the flow-through was collected. Cells were pelleted by centrifugation at low speed, followed by red blood cell lysis and 2 washes with ice-cold complete medium. Cell number and viability were determined using a hemocytometer.

For embedding, the cell pellet was resuspended in ice-cold growth factor–reduced Matrigel at the desired density and gently mixed to avoid air bubble formation. Aliquots (typically 30-50 μL) of the cell–Matrigel mixture were dispensed as domes into the center of pre-warmed wells in tissue culture plates. Plates were incubated at 37 °C for 10-15 minutes to allow Matrigel polymerization. Following polymerization, domes were overlaid with pre-warmed organoid culture medium supplemented with essential growth factors, inhibitors, and antibiotics according to established organoid culture protocols. Medium was replaced every 2-3 days, taking care to avoid disturbing the Matrigel structure.

Organoid morphology and growth were monitored by phase-contrast microscopy. Cultures were passaged when organoids reached optimal size or density, typically every 1-3 weeks, by mechanical disruption or limited enzymatic digestion followed by re-embedding in fresh Matrigel domes. All procedures were performed under sterile conditions in a biosafety cabinet, and cultures were maintained at 37 °C in a humidified incubator with 5% CO_2_.

### Immunocytochemistry and image analysis

Tissue specimens from patients and organoids were fixed in 4% paraformaldehyde for 24 hours, dehydrated through graded ethanols and xylene, and embedded in paraffin. Paraffin blocks were sectioned at 3 μm for H&E and IHC. For IHC, sections were deparaffinized, rehydrated, and subjected to heat-induced epitope retrieval. Endogenous peroxidase (0.3% H_2_O_2_) and nonspecific binding were quenched (5% normal goat serum), then incubated with following primary overnight at 4 °C: PTGS1 (Abcam, #ab109025, RRID: AB_10865291, https://scicrunch.org/resolver/AB_10865291), CD68 (Abcam, #ab303565, RRID:AB_3075482, https://scicrunch.org/resolver/AB_3075482), Vimentin (Santa Cruz Biotechnology, #sc-373717, RRID: AB_10917747, https://scicrunch.org/resolver/AB_10917747), Fibronectin (Abcam, #ab268020, RRID:AB_2941028, https://scicrunch.org/resolver/AB_2941028), Ki67 (Abcam, #ab15580, RRID:AB_443209, https://scicrunch.org/resolver/AB_443209), PRL (Abcam, #ab188229, RRID:AB_2921370, https://scicrunch.org/resolver/AB_2921370). Slides were incubated with Dako REAL™ EnVision™ HRP rabbit/mouse (K5007, DAKO, Glostrup, Denmark) at room temperature for 20 minutes, followed by applying Dako REAL™ DAB + CHROMOGEN and Dako REAL™ substrate buffer (belonging to K5007, DAKO, Glostrup, Denmark) to visualize staining signals under light microscopy, followed by hematoxylin counterstain. Slides were digitized with an Ocus scanner (Grundium, Tampere, Finland) and evaluated in QuPath 0.3.0. Image preprocessing employed QuPath's stain vector estimator; nuclei were segmented with the cell detection tool (hematoxylin channel). For each marker, positivity thresholds based on mean DAB optical density were defined from staining patterns and consistently applied to all cases. The H-score for PTGS1 was computed as the percentage of positive tumor cells multiplied by the average staining intensity (0-3). Analysts were blinded to clinical data. To mitigate outliers, H-scores were Winsorized: values below the 5th percentile were set to the 5th percentile and those above the 95th percentile were set to the 95th percentile.

### Statistical analysis

Statistical analyses were performed using SPSS 24.0 (SPSS Inc, USA) and R version 4.0.0. Continuous variables were expressed as mean ± standard deviation for normally distributed data or median (interquartile range) for non-normally distributed data, and compared using Student's t-test or Mann-Whitney U test, respectively. Categorical variables were presented as counts and proportions, and compared using chi-square test or Fisher's exact test as appropriate. Fisher's exact test was used for 2 × 2 comparisons and when expected cell frequencies were <5. Correlation analyses were conducted using Spearman's rank correlation coefficient. For multiple comparisons, the false discovery rate algorithm was applied using the Benjamini-Hochberg procedure. Two-tailed *P*-values < .05 were considered statistically significant.

## Results

### Elevated *Blautia* abundance in prolactinoma is related to invasiveness of prolactinomas

Our previous research identified a significant increase in the relative abundance of *Blautia* genus within the intestinal microbiome of individuals diagnosed with prolactinoma [31]. However, whether *Blautia* or other specific gut microbiota are associated with the biological behavior of prolactinomas remained unclear. To further explore the association between increased *Blautia* abundance in the gut and its potential influence on the biological behavior of distantly located prolactinomas, we collected both tumor and fecal samples from 10 surgically treated prolactinoma patients, comprising 4 patients with HI tumors (2 treatment-naive and 2 with prior dopamine agonist treatment) and 6 patients with LI tumors (2 treatment-naive and 4 with prior dopamine agonist treatment). All patients with prior dopamine agonist exposure had discontinued therapy due to treatment failure prior to surgery.

**Figure 1:**
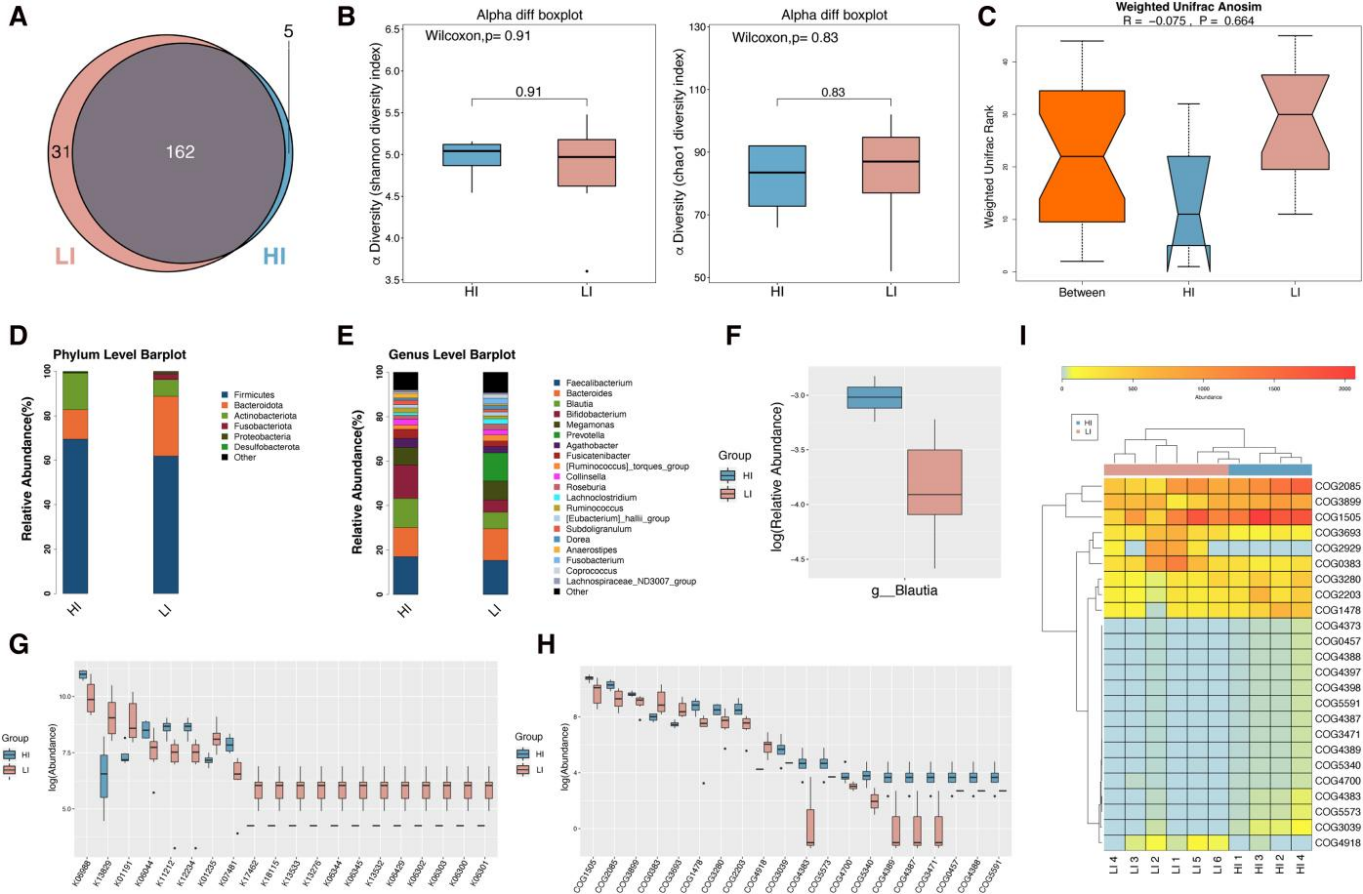
16S rRNA sequencing indicated the significant differences in the abundance of *Blautia* together with different functioning genes between high- and low-invasiveness prolactinomas. (A) Venn diagram of shared and specific OTUs in HI (*n* = 4) and LI (*n* = 6). (B) Comparison of alpha diversity represented by Shannon and Chao1 indices using Wilcoxon rank sum test between HI (*n* = 4) and LI (*n* = 6), respectively. Left: Shannon index between 2 groups (*P* = .91); Right: Chao1 index between 2 groups (*P* = .83). (C) Comparison of beta diversity based on Weighted Unifrac Anosim between HI (*n* = 4) and LI (*n* = 6) (R = −0.075, *P* = .664). (D) Barplot exhibiting relative abundance of gut microbiota at phylum level in HI (*n* = 4) and LI (*n* = 6). (E) Barplot exhibiting relative abundance of gut microbiota at genus level in HI (*n* = 4) and LI (*n* = 6). (F) Barplot showing the relative abundance of *Blautia* with a significance threshold of *P* < .05, indicating *Blautia* to be the only characteristic gut microbiota at genus level. (G) Barplots showing the abundance of top 20 KO terms with a significance threshold of *P* < .05 in HI (*n* = 4) and LI (*n* = 6) groups, respectively. (H) Barplots showing the abundance of top 20 COG terms with a significance threshold of *P* < .05 in HI (*n* = 4) and LI (*n* = 6) groups, respectively. (I) Clustered heatmap of COG functional genes enriched in HI (*n* = 4) and LI (*n* = 6) groups.

Using 16S rRNA sequencing data, we found 162 of the total 198 operational taxonomy units (OTUs) shared between the LI prolactinomas and HI prolactinomas groups. In contrast, 5 OTUs were found exclusively in the HI group (Fig. 1A). Gut microbial diversity was evaluated using α-diversity (within-sample diversity reflecting richness and evenness) and β-diversity (between-group compositional dissimilarity). Neither α-diversity nor β-diversity differed significantly between groups (Fig. 1B-1C), indicating comparable gut microbial community structure at the genus level regardless of prolactinoma invasiveness. Though the richness and evenness between the 2 groups were similar, the composition of gut microbiota differed from 2 groups. To examine these differences more closely, we investigated microbial distributions at the phylum and genus levels. Notably, the *Firmicutes* to *Bacteroidetes* (F-B ratio) was significantly higher in the HI compared to LI group (Fig. 1D). The differences were more pronounced at the genus level of taxonomic classification. Contradictory to common sense, *Blautia* and *Bifidobacterium*, 2 well-known probiotics, were more abundant in HI group than LI group, while the relative abundance of *Prevotella* surged in LI group (Fig. 1E). Further, we identified significant the gut microbiota with discrepancy between prolactinoma patients with different tumor invasiveness. Consistent with our earlier findings, *Blautia* appeared to be the only genus with significance between high- and low-invasion prolactinoma (Fig. 1F).

Next, we predicted and estimated the potential differences in protein functions and metabolic steps using 16S rRNA marker genes. In our study, we annotated gene families obtained from 16 seconds rRNA analysis using Kyoto Encyclopedia of Genes and Genomes Orthology (KO) and Cluster of Orthologous Groups (COG) database. In Fig. 1G-1I, genes related to NAD+/NADH and NADP+/NADPH circulation such as NADPH oxidoreductase (K06988), coenzyme F420-0:L-glutamate ligase/coenzyme F420-1:gamma-L-glutamate ligase (K12234) and F420-0:Gamma-glutamyl ligase (COG1478) were significantly abundant in HI prolactinomas. While the alpha-mannosidase (COG0383 and K01191), known as an enzyme capable of degradation of oncogenic N-glycans, was highly enriched in LI group.

**Figure 2:**
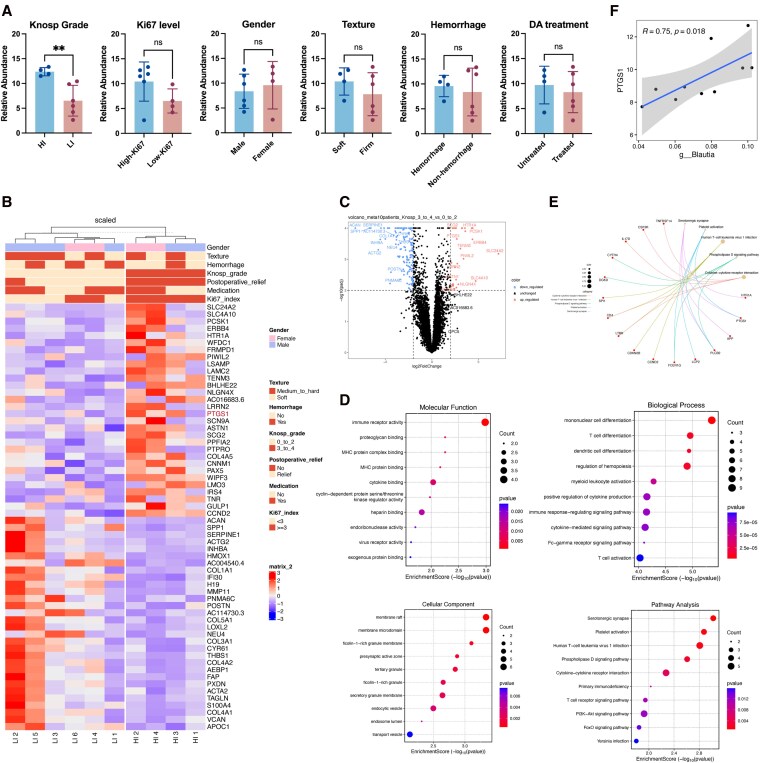
The abundance of *Blautia* and transcriptomics co-analysis of 10 prolactinoma patients. (A) Boxplot depicting the relative abundance of *Blautia* in gut microbiota from prolactinoma patients grouped by Knosp grade (HI vs LI, 12.370 ± 0.817 vs 6.522 ± 3.096, *P* = .0067), Ki67 level (High-Ki67 vs Low-Ki67, 10.420 ± 3.944 vs 6.517 ± 2.418, *P* = .1177), Gender (Male vs Female, 8.374 ± 3.454 vs 9.590 ± 4.786, *P* = .6508), Hemorrhage (Hemorrhage vs Non-hemorrhage, 9.576 ± 2.141 vs 8.383 ± 4.791, *P* = .6570), Texture (Soft vs Firm, 10.400 ± 2.747 vs 7.834 ± 4.324, *P* = .3271) and DAs treatment (Untreated vs Treated, 9.708 ± 3.772 vs 8.295 ± 4.110, *P* = .5980). **P < .01. (B) Heatmap displaying RNA-Seq expression patterns of the top 30 up-regulated and top 30 down-regulated genes in prolactinomas comparing Knosp 0-2 (low invasiveness, LI, *n* = 6) vs Knosp 3-4 (high invasiveness, HI, *n* = 4) cohorts. (C) Volcano plot identifying up-regulated and down-regulated genes in prolactinomas (HI vs LI). (D) KEGG pathway enrichment analysis and GO term analysis showing the top 10 significantly enriched pathways and biological behaviors. (FDR < 0.05). (E) Cnet plot mapping KEGG pathways (larger dots) to constituent DEGs (smaller dots). (F) PTGS1 expression scales linearly with fecal *Blautia* abundance (*R* = 0.75, *P* = .018) in prolactinomas. Solid line: regression fit; shaded band: 95% CI.

We then link the clinical characteristics with the abundance of *Blautia.* As shown in Fig. 2A, only the Knosp grade was significantly associated with the abundance of *Blautia*. Patients with more invasive prolactinomas (HI) tended to have a higher abundance of *Blautia*. No significant associations were observed between *Blautia* abundance and tumor Ki-67 index, apoplexy, tumor texture, patient gender, or dopamine agonist therapy.

### *Blautia* abundance positively correlates with elevated PTGS1 expression in prolactinomas

To explore potential molecular correlates of *Blautia* enrichment in relation to tumor invasiveness, mRNA sequencing was performed on all 10 prolactinoma samples. The heatmap in Fig. 2B compared the transcriptomic profiles of 4 highly invasive tumors (Knosp grade 3-4) with those of 6 less invasive tumors (Knosp grade 0-2), highlighting the top 30 most significantly upregulated and downregulated genes between these 2 groups.

The volcano plot in Fig. 2C revealed 437 differentially expressed genes (DEGs) (|log2FC|>2.0, *P*-value < .01) between HI and LI, with 96 genes upregulated and 341 downregulated. Notably, the oncogenes ERBB4 and PIWIL2 were upregulated in high-invasive prolactinomas. In contrast, SPP1, COL1A1, NEU4, and SERPINE1, genes associated with regulation of tumor microenvironment, were downregulated in high-invasive prolactinomas as well. KEGG (Kyoto Encyclopedia of Genes and Genomes) and GO enrichment analyses were subsequently performed to investigate the functional roles of DEGs between high- and low-invasion prolactinomas. As shown in Fig. 2D, functional annotation revealed that the DEGs were significantly enriched in immune receptor activity, proteoglycan binding and cytokine binding. In terms of biological processes, the DEGs were primarily associated with immune cell differentiation processes, particularly mononuclear cell, T cell, and dendritic cell differentiation. Pathway enrichment analysis further identified several significantly enriched signaling pathways, including serotonergic synapse, platelet activation, human T-cell leukemia virus 1 infection, phospholipase D signaling, and cytokine-cytokine receptor interaction. Integrating pathway analysis with differential gene expression data, we found that the top 2 enriched signaling pathways, serotonergic synapse and platelet activation, were both anchored at PTGS1, CCND2, HTR1A, and other immune-related genes, suggesting that alterations in immune cell ontogeny and infiltration contribute to prolactinoma invasiveness (Fig. 2E). Notably, among these genes, only PTGS1 showed a positive correlation with the relative abundance of *Blautia* (Table 1 and Fig. 2F and Fig. S1A [32]). The positive correlation between *Blautia* abundance and PTGS1 expression (*R* = 0.75, *P* = .018) suggests that gut microbiota composition may influence tumor PTGS1 levels in prolactinomas. Given PTGS1's established role in prostaglandin biosynthesis and its capacity to modulate tumor microenvironment through inflammatory mediators, we hypothesize that *Blautia*-derived metabolites or immune signals may remotely upregulate tumor PTGS1 expression, thereby reshaping the TME to promote prolactinoma invasiveness.

**Table 1:** Correlation analysis between Blautia relative abundance and DEGs in top 5 enriched signaling pathways

Genes	*R* value	*P* value		genes	*R* value	*P* value
HTR1A	0.33	.35		LTBR	0.1	.79
**PTGS1**	**0**.**75**	*.**018**		CD4	−0.39	.26
APP	0.27	.45		SPI1	−0.64	.054
PLCB2	−0.49	.15		DGKD	−0.5	.14
LCP2	−0.49	.15		CYTH4	−0.47	.18
FCER1G	−0.65	*.049		IL17D	0.1	.79
CCND2	0.48	.17		CSF3R	−0.12	.76
CDKN2B	0.54	.11		TNFRSF14	−0.54	.11

*P < .05.

### PTGS1 is a distinctive invasiveness marker for prolactinoma

**Figure 3:**
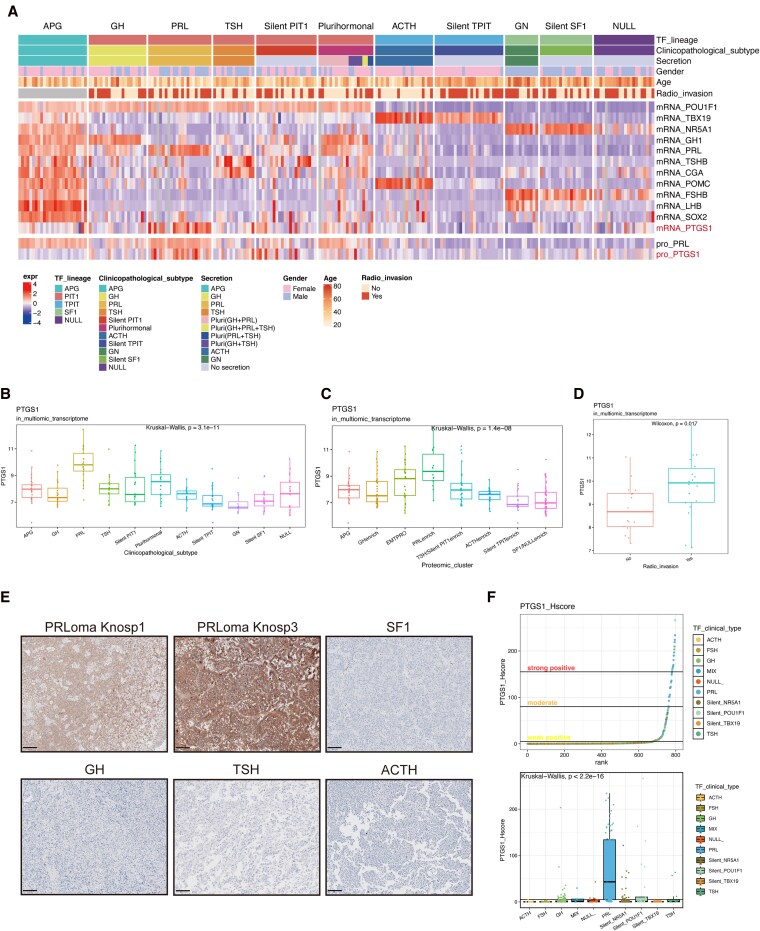
PTGS1 was an exclusive invasiveness indicator in prolactinomas in previous transcriptomics and proteomics cohort. (A) Multi-omics heatmap indicated concordant PTGS1 overexpression at mRNA and protein levels exclusively in prolactinomas. Non-PRL PitNETs show minimal expression. PTGS1 was marked in red font. (B) Boxplot depicting significantly elevated PTGS1 mRNA in prolactinomas vs other clinical pathological subtypes (*P* = 3.1e-11). (C) Boxplot depicting significantly elevated PTGS1 mRNA in PRLenrich subtype vs other proteomic subtypes (*P* = 1.4e-8). (D) Boxplot depicting significantly elevated PTGS1 mRNA in invasive prolactinomas vs noninvasive prolactinomas (*P* = .017). (E) Subtype-specific PTGS1 IHC profiling. Pronounced PTGS1 immunoreactivity exclusively in prolactinomas (Knosp grade 1 and 3) vs minimal expression in SF1, GH, TSH and ACTH. Scale bars = 100 μm. (F) TMA-based PTGS1 IHC quantification. Dot plot (top) and boxplot (bottom) validation of PTGS1 overexpression specifically in prolactinomas vs other PitNETs.

To further elucidate the role of PTGS1 in PitNETs, particularly prolactinomas, we integrated and analyzed previously generated transcriptomic and proteomic datasets from our multi-omics cohort [33]. As shown in Fig. 3A-3B, PTGS1 expression was significantly upregulated in prolactinomas at both the mRNA and protein levels, compared to normal pituitary tissues and other PitNETs subtypes (eg, somatotropinomas and corticotropinomas). Leveraging our established proteogenomic classification of PitNETs, we identified significantly elevated PTGS1 expression in the prolactin-secreting subtype. Notably, tumors exhibiting high invasiveness, larger diameter, and epithelial-mesenchymal transition (EMT) phenotypes also showed relatively high PTGS1 levels across multiple lineages. These findings suggest that PTGS1 may serve as a potential biomarker for PitNETs invasiveness, particularly in prolactinomas (Fig. 3C). Subsequent integration of clinical data confirmed significantly elevated PTGS1 expression in invasive prolactinomas (*P* = .017), supporting our initial findings from the pilot cohort of 10 patients (Fig. 3D). Immunohistochemical staining further demonstrated that PTGS1 upregulation was exclusive to prolactinomas among PitNETs (Fig. 3E). Quantitative analysis of the PitNETs tissue microarray also revealed significantly higher PTGS1 H-scores in prolactinomas (Fig. 3F). Collectively, these multi-platform analyses establish PTGS1 as a robust biomarker for prolactinoma invasiveness.

### PTGS1-high prolactinomas exhibit TME remodeling with enhanced immune infiltration and ECM alterations

**Figure 4:**
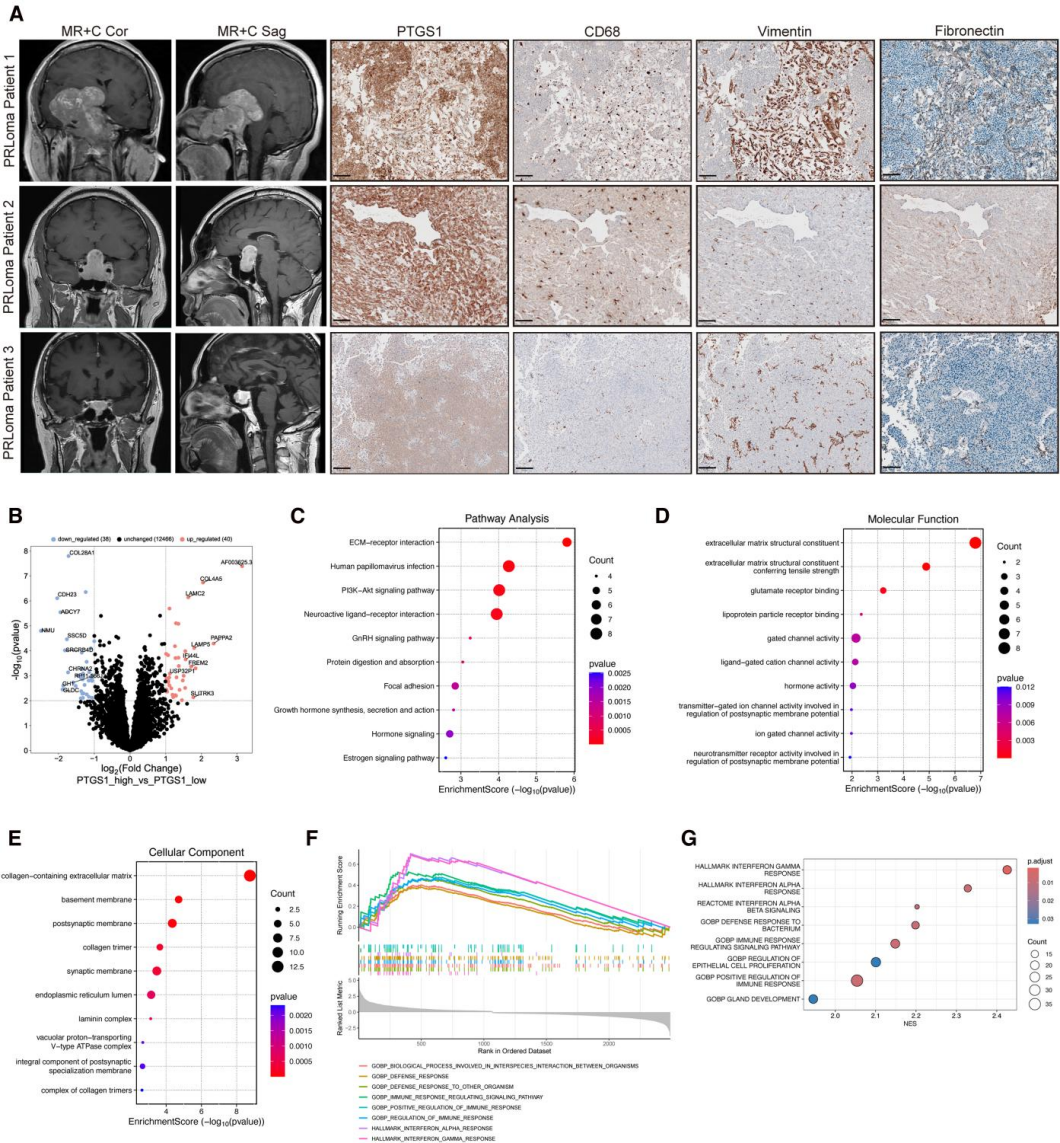
Functional enrichment landscape of PTGS1-driven transcriptomic alterations in prolactinomas. (A) Coronal and sagittal MRI image alongside corresponding tumor tissue immunohistochemical staining for PTGS1, CD68, Vimentin and Fibronectin 1 in prolactinomas with varying invasive status. Scale bars = 100 μm. (B) Volcano plot identifying up-regulated and down-regulated genes in prolactinomas with different PTGS1 expression (PTGS1-high vs PTGS1-low). (C) KEGG pathway enrichment analysis showing the top 10 significantly enriched pathways (FDR < 0.05). (D) GO molecular function enrichment showing the top 10 significantly enriched pathways (FDR < 0.05). (E) GO Cellular Component (CC) enrichment showing the top 10 significantly enriched pathways (FDR < 0.05). (F) GSEA plot revealed coordinate upregulation of immune surveillance and cell proliferation signaling pathway in PTGS1-high prolactinomas. (G) GSEA dot plot of immune surveillance and cell proliferation signaling pathway in PTGS1-high prolactinomas (NES > 1.8, *P*.adjust < .02).

Here in Fig. 4A, we demonstrate 3 representative MRI image series from prolactinoma patients with varying degrees of tumor invasiveness, illustrating PTGS1-driven pathological progression. Patient 1 exhibited a massive tumor with radiological signal heterogeneity and extensive invasion into the cavernous sinus and intracranial space, accompanied by intense PTGS1 immunoreactivity and profoundly disorganized histoarchitecture, including disordered adenoma cell arrangement, increased stromal components, enhanced infiltration of CD68-positive macrophages/monocytes within the stroma, tubular expression of vimentin, and a fibronectin network partitioning tumor cells into nest-like clusters (Fig. 4A, top row). Patient 2 showed focal cavernous sinus invasion without intracranial involvement, moderate PTGS1 expression, and a relatively preserved trabecular architecture, with scattered infiltration of CD68-positive macrophages/monocytes, microtubular vimentin expression distributed within the adenoma parenchyma, and fibronectin expression surrounding intratumoral lumina (Fig. 4A, middle row). In contrast, Patient 3 had a prolactinoma confined to the sellar region with minimal PTGS1 staining, CD68-positive cells infiltration and mild EMT phenotype (Fig. 4A, bottom row).

To elucidate transcriptomic alterations associated with differential PTGS1 expression in prolactinomas, we performed mRNA sequencing on 64 prolactinoma samples, stratifying tumors into PTGS1-high (*n* = 32, top 50% expression) and PTGS1-low (*n* = 32, bottom 50% expression) groups based on transcriptomic abundance (Table 2). As shown in Fig. 4B, comparison between PTGS1-high and PTGS1-low prolactinomas identified 78 DEGs (basemean > 100, *P*-value < .01 and |logFC|>1) with 40 up-regulated and 38 down-regulated genes. Subsequent KEGG pathway enrichment analysis of above 78 DEGs revealed significant involvement in tumor microenvironment remodeling and hormone signaling regulation. These DEGs were predominantly enriched in ECM-related signaling pathways, including ECM-receptor interaction, protein digestion and absorption and focal adhesion. Additionally, they showed strong associations with key endocrine regulatory cascades such as PI3K-AKT, GnRH, and estrogen signaling (Fig. 4C). Functional analysis of DEGs between PTGS1-high and PTGS1-low prolactinomas also revealed significant enrichment in genes involved in ECM construction. These genes were primarily related to structural components that provide strength and integrity to the ECM, and were predominantly localized to collagen-rich regions of the matrix (Fig. 4D-4E). Collectively, these findings suggest that PTGS1 expression is associated with ECM remodeling pathways implicated in prolactinoma invasiveness, consistent with our earlier pathway enrichment results.

**Table 2:** Clinical features of prolactinoma patients divided into PTGS1-high and PTGS1-low groups in this study

Characteristic	PTGS1-high (*n* = 32)	PTGS1-low (*n* = 32)	*P* value
Demographics			
Male gender, *n* (%)	23 (71.9)	18 (56.2)	0.182
Symptom course (months), median (IQR)	3.5 (1.8-12.0)	10.5 (2.0-12.0)	0.081
Tumor characteristics			
Tumor volume (cm^3^), median (IQR)	4.08 (1.37-8.24)	2.76 (0.78-6.90)	.138
Knosp grade, median (IQR)	3.0 (1.0-4.0)	1.5 (1.0-2.0)	.012
High invasion (Knosp 3-4), *n* (%)	18 (56.2)	7 (21.9)	.010
Treatment history			
Prior operation, *n* (%)	2 (6.3)	2 (6.3)	.999
Preoperative DAs treatment, *n* (%)	2 (6.3)	4 (12.6)	.673
Biochemical parameters			
Preoperative PRL (ng/mL), median (IQR)	800.9 (470.0-3973.5)	629.2 (343.0-2294.3)	.343
Postoperative PRL (ng/mL), median (IQR)	69.8 (14.0-337.4)	22.5 (10.6-135.3)	.173
Pathological features			
Ki67 index ≥3%, *n* (%)	22 (68.8)	18 (56.2)	.417
ER receptor positive, *n* (%)	26 (81.2)	28 (87.5)	.731
Treatment outcomes			
Postoperative remission, *n* (%)	13 (40.6)	16 (50.0)	.619
Adjuvant treatment for Nonremission patients			
Nonremission, *n* (%)	19 (59.4)	16 (50.0)	.619
Radiation, *n* (%)	1 (3.1)	0 (0.0)	1.000
Complementary DAs treatment, *n* (%)	14 (43.8)	13 (40.6)	.814

Abbreviations: DAs, dopamine agonists; ER, estrogen receptor; HI, high invasion (Knosp 3-4); LI, low invasion (Knosp 0-2); N/A, not applicable; PRL, prolactin.

Criteria for postoperative remission: Achievement of biochemical remission (serum prolactin <1.0×ULN measured after 6 weeks post-surgery) and/or complete clinical symptom resolution (galactorrhea cessation, sustained gonadal recovery ≥6 months).

To explore expression changes beyond conventional DEGs thresholds, we performed GSEA. As shown in Fig. 4F-4G, we observed significant activation of immune surveillance and cell proliferation pathways in PTGS1-high prolactinomas, with notable enrichment in functional axes such as interferon response, antimicrobial defense, and regulation of cell proliferation. These findings suggest that PTGS1 promotes the formation of an immune-permissive microenvironment, driving increased proliferation and invasiveness in prolactinomas. The *Blautia*-PTGS1 correlation suggests a potential gut-pituitary signaling axis. We propose that *Blautia*-derived metabolites may reach the pituitary through systemic circulation, upregulating PTGS1 and subsequently enhancing prostaglandin-dependent TME remodeling. While this represents a plausible mechanism, reverse causation and confounding factors require consideration in future studies.

### PTGS1-selective inhibitor Tenidap suppresses secretory and proliferative functions in prolactinoma organoids

**Figure 5:**
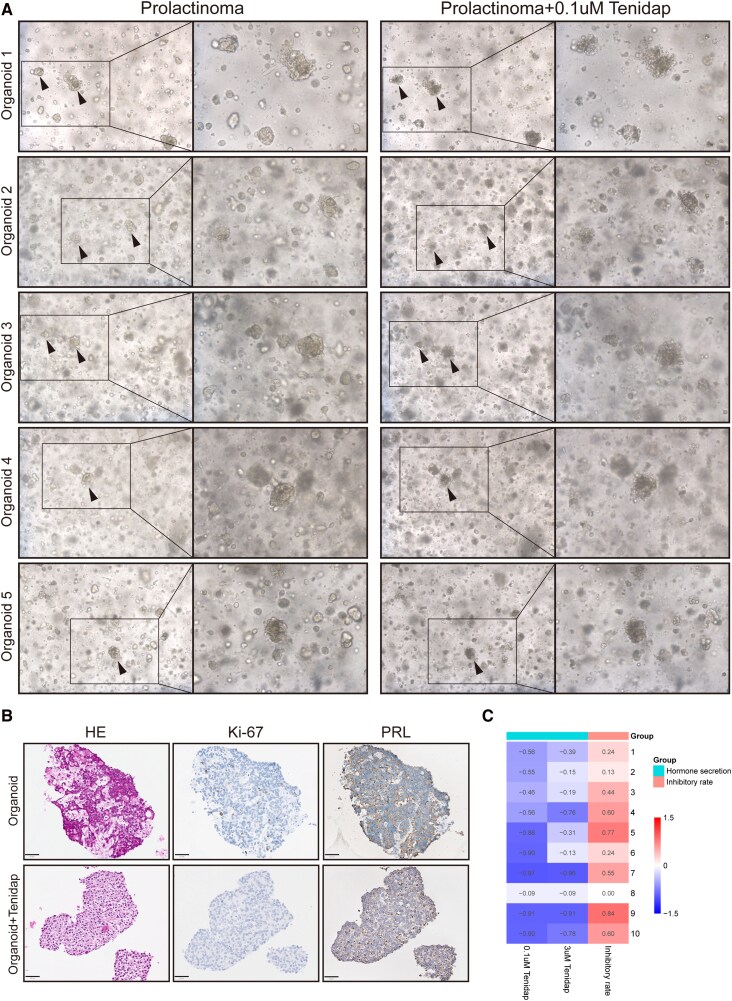
Selective PTGS1 inhibitor Tenidap exhibited an inhibitory effect on both proliferation and PRL secretion of prolactinoma organoids. (A) Representative bright-field images of 5 prolactinoma organoids. Black arrowhead indicated targeted organoids collapsed after Tenidap administration. (B) Representative histological staining of prolactinoma organoids with or without Tenidap administration. Left panel, HE staining. Middle panel, Ki-67 staining. Right panel, PRL staining. Scale bars = 50 μm. (C) Heatmap depicting the quantification of inhibitory effect of Tenidap on the secretion of PRL and the proliferation of 10 prolactinoma organoids. Left column and numbers within each cells indicated the percentage reduction in PRL concentration in the supernatant of prolactinoma organoids after 0.1 μM Tenidap administration. Middle column and numbers within each cells indicated the percentage reduction in PRL concentration in the supernatant of prolactinoma organoids after 3 μM Tenidap administration. Right column and numbers within each cells indicated the proliferation inhibitory rate of prolactinoma organoids after 0.1 μM Tenidap administration.

To assess the therapeutic potential of PTGS1 inhibition, 5 patient-derived prolactinoma organoids were treated with the selective PTGS1 inhibitor Tenidap at a concentration of .1 μM. As shown in Fig. 5A, Tenidap treatment led to pronounced morphological changes, including organoid disintegration and structural collapse. Hematoxylin and eosin staining, along with immunohistochemical analysis of Ki-67 and PRL, demonstrated that PTGS1 inhibition directly reduced tumor cell proliferation and hormone hypersecretion (Fig. 5B). Quantitative analysis of the supernatant, as shown in the heatmap in Fig. 5C, revealed that Tenidap treatment significantly reduced PRL concentrations in prolactinoma organoids compared to pretreatment levels. Concurrently, CellTiter viability assays demonstrated a dose-dependent inhibitory effect, with low-dose Tenidap (0.1 uM) producing a stronger suppression of cell viability than the higher dose (3 uM). Taken together, selective PTGS1 inhibition with low-dose Tenidap resulted in greater suppression of prolactin secretion and organoid viability compared with the high dose, demonstrating paradoxical efficacy that may be mediated through PTGS1-specific mechanisms.

## Discussion

Building on previous findings of gut microbiota alterations in prolactinoma, our study integrated 16S rRNA sequencing data and clinical profiles from prolactinoma patients [31]. Our study revealed that elevated *Blautia* abundance in prolactinoma patients was correlated with higher Knosp grades. Transcriptomics identified concomitant PTGS1 upregulation in Knosp 3-4 tumors, where PTGS1 expression positively correlated with *Blautia* levels, suggesting a potential microbiota-PTGS1 association. Multi-omics analysis of 200 PitNETs confirmed exclusive PTGS1 upregulation in prolactinomas, especially those with cavernous sinus invasion. In a validation cohort, PTGS1-high tumors were enriched for pathways related to ECM remodeling and immune dysregulation. Finally, the selective PTGS1 inhibitor Tenidap suppressed prolactin secretion and viability in patient-derived organoids, supporting PTGS1 as a therapeutic target.

The gut microbiota is a complex ecosystem that maintains a mutually beneficial relationship with its host [34]. Previous studies have demonstrated a relationship between the gut microbiota and various types of PitNETs, including prolactinomas, somatotropinomas, Cushing's disease, and other nonfunction pituitary adenomas [26, 27, 31, 35]. The structural basis for the connection between gut microbes and the central nervous system, also known as gut-brain axis, can be attributed to at least 3 mechanisms: neural, endocrine, and immune signaling [36]. Although few studies have explored the remote regulatory effect of the gut microbiota on PitNETs, Nie et al demonstrated that fecal microbiota transplantation in GH-secreting pituitary adenoma led to increased infiltration of CD4 + and CD8+ T cells, along with the elevated PD-L1 expression [37]. Lie et al pioneered an integrated analysis of gut microbiome and serum metabolome, identifying a *Blautia*-serum metabolite regulatory axis that promotes aggressiveness in nonfunctioning PitNETs [38]. *Blautia* is known as a genus of anaerobic bacteria with probiotic properties, commonly found in the intestines and feces of mammals [39]. The final products of glucose fermentation by *Blautia* include acetic acid, succinic acid, lactic acid, and ethanol. Wang et al discovered 2 species from *genus Blautia*, *Blautia coccoides*, and *Blautia obeum*, that are capable of inducing type I interferon (IFN-I) responses in macrophages via the MAVS-IRF3-IFNAR signaling pathway [40]. Research on tumorigenesis has demonstrated that *Blautia* exerts a dual regulatory effect on the infiltration of CD8 + T cells within the tumor microenvironment (TME) [41, 42]. Our previous data demonstrated a significant enrichment of *Blautia* in hosts with prolactinomas [31]. Further analysis of the cohort revealed a significant difference in the relative abundance of *Blautia* among prolactinomas with different Knosp grades, suggesting its potential as a biomarker for invasive prolactinoma. Building on established evidence that *Blautia* modulates the TME through immunoregulatory pathways, we propose a plausible mechanism by which *Blautia* remodels the prolactinoma TME to promote invasion.

The correlation between *Blautia* abundance and tumor transcriptional profiles raises the possibility that gut microbiota composition may be associated with gene expression patterns in prolactinomas, though the nature and direction of this relationship require further investigation. Elucidating these microbiota-gene regulatory cascades, particularly within the gut-pituitary axis, is critical for understanding how distal microbial shifts can orchestrate sellar tumor invasion. Based on the observed correlation between *Blautia* enrichment and PTGS1 expression in prolactinomas, we hypothesize a potential *Blautia*-PTGS1 association that may be linked to TME alterations in prolactinomas. However, the mechanistic relationship, directionality, and role of potential confounding factors remain to be established. PTGS1, also known as COX-1, was has been highly expressed in breast, ovarian epithelial, and colon cancer cells [43, 44]. Previous research has delineated a PTGS1-centered immunosuppressive circuit during the resolution of inflammation, in which IFNγ induces macrophage PTGS1 expression, leading to sustained prostaglandin E2 (PGE2) biosynthesis. This process suppresses local innate immunity and lymphocyte function while promoting the generation of myeloid-derived suppressor cells, thereby establishing PTGS1 as a pivotal orchestrator of immune tolerance during inflammatory resolution [45]. Recent studies have reported that PTGS1, as well as its therapeutic targeting with commonly used COX-1/2 inhibitors, exhibits anti-tumor effects. Yang et al demonstrated a critical anti-metastatic function of aspirin and COX-1 inhibitors, showing that these agents enhance the anti-tumor activity of T cells by limiting platelet TXA2-induced immune suppression [46]. High-throughput small-molecule drug screening demonstrated that inhibition of COX-1/2, in combination with αPD-L1 therapy, suppressed tumor growth in a CD8+ T cell-dependent manner both *in vivo* and *in vitro* [47]. The bioactive compound dihydroartemisinin inhibits nonsmall cell lung cancer by silencing PTGS1 through increased ROS production and activation of ER stress, as well as the JNK and p38 MAPK signaling pathways [48].

Previous research has provided an inflammatory basis for prolactinoma development. Wang et al identified that activation of the microglial NLRP3 inflammasome leads to upregulation of the inflammatory cytokines and promotes the formation of prolactinomas [49]. In cabergoline-treated prolactinomas, Zhang et al observed an increased number of CD8+ T cells expressing cytotoxic granule components such as perforin, the granzymes GZMB and GNLY, KLRD1, as well as the inflammatory cytokine CCL5 [50]. Moreover, Szabó et al [51] elucidated the molecular mechanisms by which aspirin, a nonselective COX inhibitor, regulates global DNA demethylation, p53 acetylation, and Pttg1 expression in PitNETs. In our study, we successfully distinguished the TME regulation based on PTGS1 differently expression. DEGs between the PTGS1-high and PTGS1-low groups were primarily enriched in TME-related pathways, particularly immune regulatory signaling such as interferon response pathways and response to bacterium. Pathway enrichment analysis of these DEGs revealed transcriptomic alterations in PTGS1-high tumors, providing correlative evidence consistent with a potential *Blautia*-PTGS1-TME association. These pathways are known to be involved in ECM remodeling and immune regulation, processes relevant to tumor invasiveness, though causal relationships remain to be established. To validate the potential therapeutic effect targeting PTGS1, we established patient-derived prolactinoma organoids from 5 patients in our initial cohort and demonstrated that the selective PTGS1 inhibitor Tenidap effectively suppressed both tumor cell viability and prolactin secretion. Notably, 3 of these 5 patients had experienced preoperative DAs resistance or intolerance necessitating surgical intervention. Critically, PTGS1 inhibition via Tenidap effectively suppressed tumor growth and prolactin secretion in organoids derived from DAs-refractory patients, demonstrating therapeutic efficacy in this clinically challenging population. These findings suggest that PTGS1 represents a promising complementary or alternative therapeutic target for prolactinoma patients who are refractory to conventional DAs therapy.

This study has several limitations. The small sample size constrains statistical power, and while confounders can be controlled by statistical analysis, the *Blautia*-invasiveness association represents correlation rather than causation. The mechanistic pathway linking *Blautia* to PTGS1 expression remains undefined—whether mediated by microbial metabolites or immune factors is unknown. Additionally, our organoid studies lacked head-to-head comparison with dopamine agonists, limiting therapeutic context. Future work should pursue following complementary directions. First, validation in larger cohorts with comprehensive phenotyping using multivariate statistical frameworks. Second, mechanistic experiments in germ-free animal models to establish causality, testing whether *Blautia* manipulation affects tumor invasiveness and PTGS1 expression, and identifying the molecular mediators through multi-omics approaches. Third, translational studies comparing and combining PTGS1 inhibitors with standard therapies in preclinical models. Prospective studies tracking microbiome dynamics during disease progression would further clarify temporal relationships. These integrated approaches are essential to determine whether *Blautia* represents a causal driver, biomarker, or therapeutic target.

In conclusion, we integrated multi-omics data from prolactinoma cohorts and identified associations between gut microbiota composition and tumor gene expression patterns. 16S rRNA and mRNA sequencing revealed a positive correlation between *Blautia* abundance and tumor PTGS1 expression in a pilot cohort. Multi-cohort transcriptomic and proteomic analyses confirmed PTGS1 upregulation specifically in prolactinomas, with PTGS1-high tumors showing enrichment of TME remodeling and immune-related pathways. These findings establish correlative links between gut microbiota, PTGS1 expression, and tumor invasiveness in prolactinomas. Our results generate hypotheses regarding potential gut-brain communication involving *Blautia*, PTGS1, and the tumor microenvironment, which warrant mechanistic validation through interventional studies to determine causality and directionality.

## Data Availability

Some or all datasets generated during and/or analyzed during the current study are not publicly available but are available from the corresponding author on reasonable request.

## Availability of data and materials

The RNA sequencing data generated in this study have been deposited in the OMIX database (https://ngdc.cncb.ac.cn/omix) under accession number OMIX015117.
